# Patient‐derived xenograft mouse models of pseudomyxoma peritonei recapitulate the human inflammatory tumor microenvironment

**DOI:** 10.1002/cam4.640

**Published:** 2016-02-02

**Authors:** Murali R. Kuracha, Peter Thomas, Brian W. Loggie, Venkatesh Govindarajan

**Affiliations:** ^1^ Department of SurgeryCreighton University2500 California PlazaOmahaNebraska68178; ^2^ Department of Biomedical SciencesCreighton University2500 California PlazaOmahaNebraska68178

**Keywords:** Ascites, chemokines, cytokines, model, mouse, peritonei, pseudomyxoma, xenograft

## Abstract

Pseudomyxoma peritonei (PMP) is a neoplastic syndrome characterized by peritoneal tumor implants with copious mucinous ascites. The standard of care for PMP patients is aggressive cytoreductive surgery performed in conjunction with heated intraperitoneal chemotherapy. Not all patients are candidates for these procedures and a majority of the patients will have recurrent disease. In addition to secreted mucin, inflammation and fibrosis are central to PMP pathogenesis but the molecular processes that regulate tumor‐stromal interactions within the peritoneal tumor microenvironment remain largely unknown. This knowledge is critical not only to elucidate PMP pathobiology but also to identify novel targets for therapy. Here, we report the generation of patient‐derived xenograft (PDX) mouse models for PMP and assess the ability of these models to replicate the inflammatory peritoneal microenvironment of human PMP patients. PDX mouse models of low‐ and high‐grade PMP were generated and were of a similar histopathology as human PMP. Cytokines previously shown to be elevated in human PMP were also elevated in PDX ascites. Significant differences in IL‐6 and IL‐8/KC/MIP2 were seen between human and PDX ascites. Interestingly, these cytokines were mostly secreted by mouse‐derived, tumor‐associated stromal cells rather than by human‐derived PMP tumor cells. Our data suggest that the PMP PDX mouse models are especially suited to the study of tumor‐stromal interactions that regulate the peritoneal inflammatory environment in PMP as the tumor and stromal cells in these mouse models are of human and murine origins, respectively. These mouse models are therefore, likely to be useful in vivo surrogates for testing and developing novel therapeutic treatment interventions for PMP.

## Introduction

Pseudomyxoma peritonei (PMP) is a mucinous neoplasm that is characterized by multifocal, tumor implants in the peritoneal cavity with excessive accumulation of “jelly‐like” ascites 1. PMP arises from a mucinous appendiceal neoplasm that spreads to the peritoneal cavity by appendiceal rupture. The tumor implants grow along the peritoneal surface often involving the omentum and visceral serosal surface. Hematogenous and nodal metastases are extremely rare. PMP is an uncommon secondary peritoneal surface malignancy 2.

PMP is biologically heterogeneous and can range from indolent tumors with excessive production of ascites to aggressive tumors with high‐grade pathology similar to those seen in peritoneal carcinomatosis of colorectal origin. Ronnett et al. classified PMP into three categories correlating histological grade with disease severity: (1) disseminated peritoneal adenomucinosis (DPAM) consisting of peritoneal lesions with bland histology and abundant mucin, (2) peritoneal mucinous carcinomatosis (PMCA) consisting of abundant gland‐forming mucinous epithelium with cytological atypia, and (3) PMCA intermediate/discordant with predominantly bland histology but with focal areas of well‐differentiated mucinous carcinoma 3. Subsequent schemes classified PMP into a two tiered system of high and low grades 4. We have recently proposed a new system with three categories that correlate well to disease outcomes: (1) PMP1 (similar to DPAM), (2) PMP2 (an intermediate category that includes cases that do not belong to PMP1 or PMP3), and (3) PMP3 that exclusively contain cases with any number of signet cells 5. Median survival for PMP1, PMP2, and PMP3 was 120, 88, and 40 months, respectively, and 5‐year survival rates were 85%, 63%, and 32%, respectively 5. These three categories are concordant with the recently published consensus that classifies PMP into three categories; low grade, high grade, and high grade with signet ring cells.

PMP can be fatal if left untreated. But treatment options for PMP patients are limited. Aggressive surgical cytoreduction in conjunction with hyperthermic intraperitoneal chemotherapy (perfusion of chemotherapeutic agents within the peritoneal cavity at 42°C) is the only treatment option that has been showed to improve patient outcomes 6, 7, 8, 9. This underscores the need for development of more effective, and less toxic, alternative treatment approaches. For this, preclinical in vitro and in vivo models are essential. Genetically engineered mouse models for PMP have not been generated (presumably due to a lack of a gene promoter specific to, or active in, appendiceal goblet cells that could drive expression of oncogenes or cre‐recombinase for conditional deletion of gene of interest). An alternative to these autochthonous mouse models are human cell‐line‐derived and PMP patient‐derived xenograft (PDX) rodent models. Cell‐line‐derived xenografts have been generated by intraperitoneal injection of cell lines with a “PMP‐like” phenotype (in the absence of an established PMP cell line) and growth of “PMP‐like” tumors in nude mice 10. PDX models are generated by serial grafting of patient‐derived tumors in immune compromised mice. The grafted tumors have been shown to maintain genetic and phenotypic heterogeneity of the original human tumors and recreate the natural course of tumor progression 11 and are therefore, viewed as more useful preclinical surrogates than genetically engineered mouse models for testing potential anticancer drugs. Recently, PMP PDX models that replicate the histopathology of the original human PMP tumors have been reported in mice 12, 13 and in rats 14. These reports describe successful xenografting of high grade, but not low grade, PMP tumors. The focus of these reports has been on the characterization of gross morphology and histopathology of the PDX PMP tumors. The nature of tumor–host interactions within the peritoneal tumor microenvironment were not explored in these studies.

In our previous studies, we have measured the levels of several chemokines and cytokines in PMP patient ascites and sera 15. These studies revealed that the chemokine/cytokine profile of the peritoneal tumor milieu was distinct from those associated with infection‐ or injury‐associated inflammation. In this study, we report the generation of low‐ and high‐grade PMP PDX models. We further show that these PDX models show a similar chemokine/cytokine expression profile as the original human tumors and therefore, replicate the molecular characteristics of the human peritoneal microenvironment. Our studies suggest that the human versus mouse species differences that exist in the PDX mouse models can be exploited to investigate tumor–stromal interactions that help sustain the inflammatory tumor microenvironment in PMP.

## Materials and Methods

### Tumor samples

Discarded tumor specimens and ascites collected from PMP patients at the time of surgery were used for this study. Collection of these samples was approved by the Creighton Institutional Review Board and all patients provided written consent for participation in research.

### Generation of PMP PDX models

All the mouse studies described here were approved by the Institutional Animal Care and Use Committee at Creighton University. PMP tumors removed at the time of surgery were washed in sterile phosphate‐buffered saline, finely chopped, mechanically disrupted by passing through an 18G needle several times and suspended in saline. This suspension was then injected intraperitoneally into 3–4 homozygous female nude mice (Crl:NU (NCr)‐Foxn1^nu^) (Charles River labs; Wilmington, MA). Mice were monitored for tumor growth by assessment of increase in girth or body weight. After ~2–6 months, mice were sacrificed, dissected and intraperitoneal tumors and ascites were collected for subsequent passage. Tumors from multiple mice were pooled to reduce clonal variance and were serially passaged in nude mice by intraperitoneal injections as described above. After successful passaging for 3–5 generations, tumor samples were harvested, preserved in Roswell Park Memorial Institute (RPMI) 1640 (Hyclone Labs, Logan, UT) complete medium containing 10% fetal bovine serum and penicillin/streptomycin and frozen in liquid nitrogen for future passage. At each passage, gross invasion of organs and spread of cancer beyond the diaphragm were assessed.

### Histology

PMP tumors from PDX mice were harvested, fixed in 10% neutral‐buffered formalin, dehydrated, embedded in paraffin, sectioned (5–7 *μ*m), and stained with hematoxylin and eosin.

### Analysis of cytokines

Alterations in cytokine/chemokine levels in human and mouse PDX ascites and sera were assessed using the Milliplex MAP kit (EMD Millipore Corporation, Billerica, MA). Human cytokines that were assayed using anti‐human and anti‐mouse antibodies are listed in Table 2. The Luminex assay was performed following manufacturer's instructions. Briefly, microspheres containing two fluorescent dyes and coated with a specific capture antibody directed against a target cytokine were used. These beads were then incubated with our samples overnight, at 4°C to allow the capture antibody to bind to the target analyte. This antigen–antibody complex was then incubated with a biotinylated detection antibody for an hour at room temperature. A streptavidin‐phycoerythrin conjugate that binds to the biotinylated detection antibody was then used as a reporter to detect the antigen–antibody complex. The microspheres were passed through a bead analyzer (Luminex 200, Luminex, Madison, WI) that includes a dual laser system with one laser activating the fluorescent dye within the beads (identifying the specific analyte) and a second laser exciting the streptavidin‐phycoerythrin conjugate bound to the beads with an emission proportionate to the concentration of the analyte. Concentrations of all cytokines reported in this study are in pg/mL.

### Immunohistochemistry

**Figure 1:**
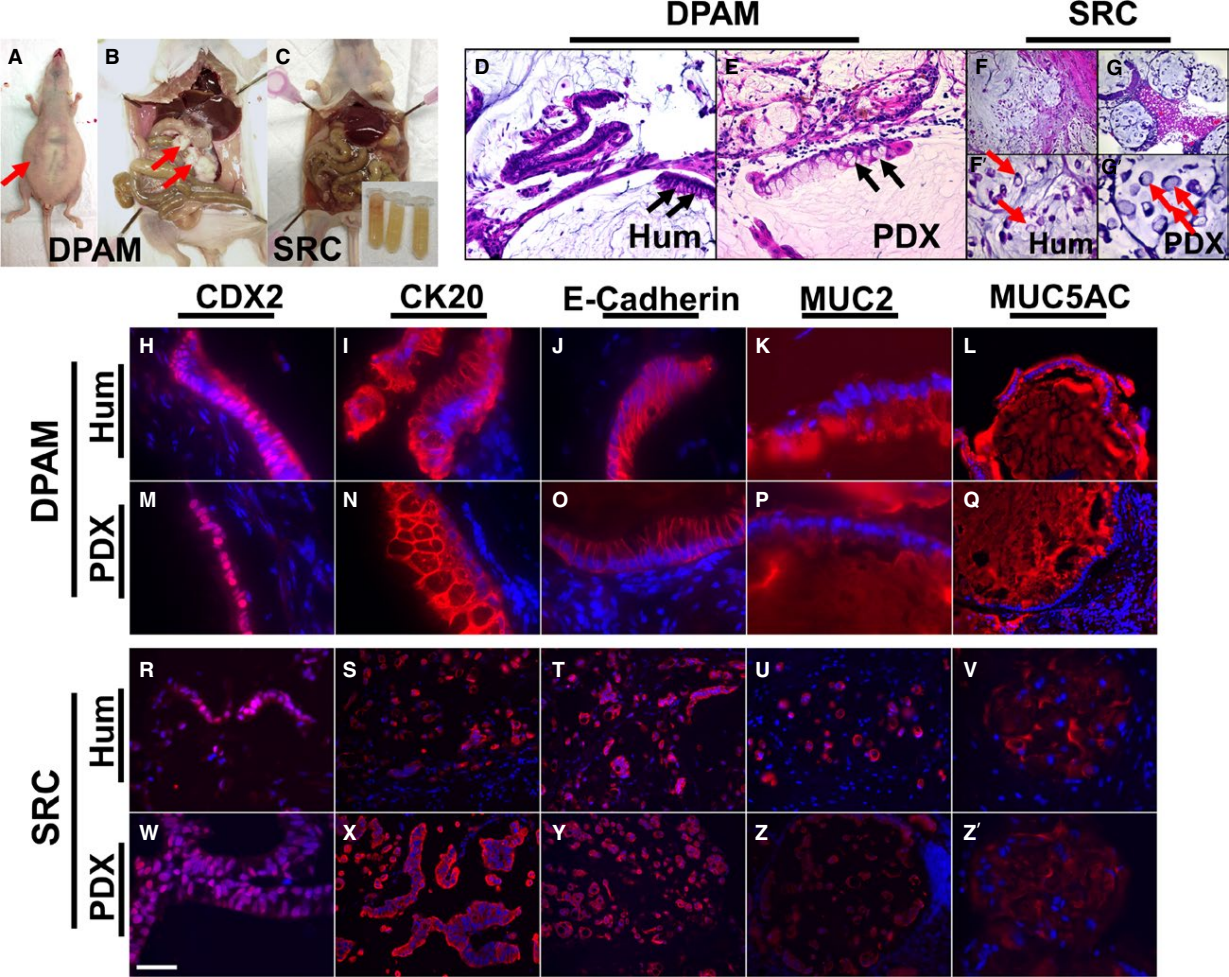
Gross morphological (A–C), histological (D–G’), and immunohistochemical (H–Z’) characteristics of PMP tumors in PDX models were similar to human PMP. Arrows in (A and B) point to a distended abdomen filled with ascites and tumor implants on serosal surface of visceral organs respectively. Arrows in (D and E) point to goblet cells. Arrows in (F’ and G’) point to signet ring cells. Inset in (C) (bottom right) shows tubes filled with ascites collected from the mouse. In (H–Z’), antigen‐antibody complexes are in red and nuclei are stained blue with DAPI. DPAM, disseminated peritoneal adenomucinosis; Hum, human; PDX, patient‐derived xenograft; SRC, signet ring cells. Scale bar: 60 *μ*m in (F and G); 30 *μ*m in (D, E, L, Q, S–U, X–Z); 15 *μ*m in (F’, G’, R, W, V, and Z’); 10 *μ*m in (H, I, J, K, M–P).

Immunohistochemistry (IHC) on formalin‐fixed, paraffin‐embedded sections was performed as described previously 16. The following antibodies were used; CDX2 (catalog number ab76541, 1:250; Abcam, Cambridge, MA), CK20 (catalog number ab76126, 1:100; Abcam, Cambridge, MA), E‐Cadherin (catalog number 610181, 1:1000; BD Biosciences, Franklin Lakes, NJ) Mucin 2 (MUC2) (catalog number 555926, 1:100; BD Biosciences, Franklin Lakes, NJ), MUC5AC (catalog number MS‐551‐P1, 1:100; Thermo Lab Vision, Fremont, CA), IL6 (catalog number BS0782R, 1:100; Bioss, Woburn, MA), KC (catalog number NBP1‐46392, 1:100; Novus, Littleton, CO), and MCP1 (catalog number BS1101R, 1:100; Bioss, Woburn, MA). Sections were mounted using antifade medium containing DAPI (ProLong, Invitrogen, Carlsbad, CA). In Figure 1 where IHC data are shown, antigen–antibody complexes are in red and nuclei are stained blue with DAPI.

### Statistical analysis

Cytokine and chemokine expression levels in ascites and sera from PMP patients and PDX models were compared using GraphPad Prism 5.04 (GraphPad Software Inc., La Jolla, CA). Cytokines and chemokines that were measured showed a broad range of concentrations in ascites and in sera and therefore, in order to minimize outlier effects, the median, instead of the mean, was used as a measure of central tendency. Also, as the normality of distributions could not be ascertained, we have taken a conservative approach and used the nonparametric Mann–Whitney test (which compares means of rank sums) to assess significance. A *P* < 0.05 (two tailed) was considered significant. Median values with interquartile ranges have been plotted on the graphs.

## Results

### PMP PDX tumors: histopathology

PMP PDX mouse models were generated by intraperitoneal injections of human PMP tumor samples resected at the time of surgery (Table 1). Thirteen human PMP samples were grafted in nude mice and of these, six could be serially passaged for at least three generations. These six PDX models were analyzed and of these one was of high grade (PMP3 with signet cells) and the other five were of low grade (PMP1 or DPAM). PMP2 tumors were not grafted due to lack of availability of specimens. The mice carrying PMP1 or PMP3 tumors typically presented with abdominal distension (Fig. 1A, arrow), a gelatinous morphology with mucinous excrescence (Fig. 1C) or presence of tumor nodules on serosal surface of visceral organs (Fig. 1B, arrows). However, no serosal invasion or spread beyond the diaphragm was seen. The PDX tumors (Fig. 1E, G, and G') were histologically similar to the original human tumors (Fig. 1D, F, and F'). Similar to human PMP (Fig. 1D), PMP1 (or DPAM) PDX tumors showed scant tumor epithelia with goblet cells (Fig. 1E, arrows) and bland histology. Similar to signet ring human PMP (Fig. 1F and F', arrows), signet ring cells were seen in high‐grade PMP3 PDX mouse tumors (Fig. 1G, and G', arrows). The PMP PDX tumors expressed proteins that have been shown to be enriched in (but not restricted to) human PMP tumors such as caudal type homeobox 2 (CDX2), cytokeratin 20 (CK20), E‐Cadherin, MUC2, and Mucin 5AC (MUC5AC) (Fig. 1H–Z’) 17, 18, 19. CDX2, a homeodomain transcription factor, has been shown to be critical for MUC2 expression in intestinal goblet cells 18, 20. CK20, an intermediate filament protein, and E‐Cadherin, the cell adhesion protein, have been shown to be expressed in PMP tumors 17, 19. A characteristic feature of PMP is the overexpression of mucins including MUC2 and MUC5AC 1. Immunohistochemical analyses revealed a similar expression and distribution patterns of CDX2 (nuclear, Fig. 1H, M, R, and W), CK20 (cytoplasmic, Fig. 1I, N, S, and X), E‐Cadherin (membrane, Fig. 1J, O, T, and Y), MUC2 (extracellular, [Fig. 1K and P]; intracellular signet ring PMP [Fig. 1U and Z]) and MUC5AC (extra‐ and intracellular, Fig. 1L, Q, V, and Z') in PMP PDX tumor as in human tumors. Taken together, these results suggested that our PMP PDX mouse models recapitulated the gross morphology and histopathology of human PMP tumors.

**Table 1:** Clinical information on tumor samples

PMP grade	CCR^a^	Previous clinical history	Prior chemotherapy	PCI score
PMP3 (high‐grade PMP with signet cells)^b^	R2C	Right hemicolectomy/HIPEC	FOLFIRI, Avastin, chemo	>20
PMP1 (low‐grade PMP or DPAM)	R1	Prior debulking	No chemotherapy	<16
PMP1 (low‐grade PMP or DPAM)	R1	No prior treatment	No chemotherapy	<16
PMP1 (low‐grade PMP or DPAM)	R1	No prior treatment	No chemotherapy	<16
PMP1 (low‐grade PMP or DPAM)	R2C pelvis	Multiple debulking	Partial debulking for symptoms could not tolerate chemotherapy	>20
PMP1 (low‐grade PMP or DPAM)	R1	Prior debulking	No chemotherapy	<16

PMP, pseudomyxoma peritonei; CCR, completeness of cytoreduction; DPAM, disseminated peritoneal adenomucinosis; PCI, peritoneal cancer index.

a The level of cytoreduction was scored as follows: R1, no visible disease; R2a, residual tumor nodules ≤5 mm; R2b, residual tumor nodules >5 mm but ≤2 cm; R2c, residual tumor nodules >2 cm; and R3, unresectable tumor nodules.

b Initial pathology was DPAM (low‐grade PMP or PMP1) and lymph node negative; cytoreductive surgery with hyperthermic intraperitoneal chemotherapy; recurred with signet cells.

### Comparison of PMP human and PDX cytokine/chemokine profiles

Our previous studies showed that the cytokines IL‐6, IL‐8, IP‐10, MCP‐1, and MIP1*α* were significantly elevated in PMP patient ascites 15. In order to test whether the peritoneal tumor microenvironment of our PDX mouse models shows a similar cytokine expression profile, we measured the expression levels of 17 cytokines/chemokines in ascites collected from PMP PDX models and PMP patients (Table 2). The multiplex assay for measurement of cytokines was performed using anti‐human antibodies. Of the 17 cytokines/chemokines, only IL6, GRO, and IL8 levels were significantly different (*P* < 0.05) between PMP human and PDX mouse ascites (Table 3). Median levels of all these three cytokines were significantly elevated in human compared to PDX ascites IL6 (897‐fold), GRO (~70‐fold) and IL8 (~50‐fold). These results suggested that a majority of the cytokines that were tested were produced in PMP PDX models at levels comparable to those in PMP patient ascites.

**Table 2:** List of chemokines and cytokines measured in this study

	Cytokine/chemokine	Full name	Cytokine designation
1	Eotaxin^a^	Eotaxin	CCL11
2	Flt‐3L^a^	Fms‐like tyrosine kinase‐3 Ligand	
3	GRO^a^	Growth‐regulated alpha protein	CXCL1
4	IFN*α*2^a^	Interferon alpha 2	
5	INF*γ* b	Interferon gamma	
6	IP‐10^b^	Interferon‐inducible protein‐10	CXCL10
7	IL‐10^b^	Interleukin 10	
8	IL‐6^b^	Interleukin 6	
9	IL‐8^b^	Interleukin 8	CXCL8
10	IL‐1r*α* a	Interleukin‐1 receptor antagonist protein	
11	MIP‐1*α* b	Macrophage inflammatory protein 1‐alpha	CCL3
12	MIP‐1*β* b	Macrophage inflammatory protein 1‐beta	CCL4
13	MDC^a^	Macrophage‐derived chemokine	CCL22
14	MCP‐1^a^	Monocyte chemoattractant protein 1	CCL2
15	sCD‐40L^a^	Soluble CD40‐ligand	
16	TNF‐*α* b	Tumor necrosis factor	
17	VEGF^b^	Vascular endothelial growth factor	

a Cytokine levels were measured using anti‐human antibodies.

b Cytokine levels were measured using anti‐human and anti‐mouse antibodies.

**Table 3:** Comparison of cytokine/chemokine levels between human and PDX ascites using anti‐human antibodies

Cytokine/chemokine	Human PMP ascites, median (range)	PDX PMP ascites, median (range)	Significance
IP‐10	1610 (27.98–4883)	18.52 (0.39–4945)	ns
IL‐6	915 (268–9261)	1.02 (1.02–124)	0.0067
MCP‐1	838 (188–10,443)	30.35 (2.13–5200)	ns
MDC	369 (3.05–681)	7.89 (3.05–120)	ns
sCD‐40L	305 (6.2–641)	18.69 (6.2–2265)	ns
GRO	263 (191–556)	3.77 (3.77–493)	0.0316
IL‐8	195 (107–938)	3.925 (1.19–533)	0.0341
VEGF	103 (9.14–3792)	2515 (1175–9850)	ns
Eotaxin	56.88 (27.3–582)	18.68 (3.59–80.96)	ns
IL‐10	56.21 (1.66–337)	1.66 (1.66–36.89)	ns
IL‐1r*α*	22.93 (1.82–30.67)	1.82 (1.82–1644)	ns
MIP‐1*β*	18.63 (0.96–43.52)	0.96 (0.96–87.7)	ns
TNF‐*α*	9.09 (1.25–16.21)	1.25 (1.25–19.65)	ns
MIP‐1*α*	7.44 (2.03–30.92)	2.03 (2.03–13.43)	ns
IFN*α*2	5.1 (0.44–49.58)	0.44 (2.14–86.39)	ns
IFN*γ*	3.02 (1.51–8.14)	5.64 (1.51–51.15)	ns
Flt‐3L	2.14 (2.14–168)	2.14 (2.14–86.39)	ns

ns, not significant.

In order to analyze the reasons for increased expression of GRO, IL6, and IL8 in human but not in PDX ascites, we considered the possibility that these cytokines were secreted by tumor‐associated stromal cells. As these cells are likely to be of murine origin in the PDX models, we reasoned that the human antibodies that were used for the multiplex assays were unlikely to cross react with, and therefore, unlikely to detect mouse GRO, IL6, and IL8 antigens. In order to test this hypothesis, we repeated the multiplex assay on mouse PDX ascites using anti‐mouse antibodies and measured the expression levels of 10 cytokines including mouse homologs of IL‐6 and IL‐8 (Table 4). Mice do not express GRO or IL8, and KC (encoded by the CXCL1 gene) and MIP2 (encoded by the CXCL2 gene) are the closest homologs of human IL8 21, 22, 23. Significant differences between human and PDX ascites in IL‐6 and IL‐8/KC/MIP2 expression levels (Table 4) were still observed. However, the fold difference in median expression levels of IL‐6 had reduced from 897‐fold (915 pg/mL in human vs. 1.02 pg/mL in PDX, Table 3) to 6.4‐fold (915 pg/mL in human vs. 142.5 pg/mL in PDX, Table 4). Similar reduction were seen in the levels of other cytokines including IP10, IL8/KC, IL8/MIP2, IL10, MCP1, MIP1*β*, TNF*α*, and MIP1*α* (Table 5). These results support the notion that these cytokines in the PMP PDX models were secreted by murine cells and are therefore, likely from the tumor‐associated stroma. In contrast, the increase in fold difference in median IFN*γ* and VEGF expression levels (Table 5) suggested that these cytokines were mostly generated by cells of human origin that is, PMP tumor cells.

**Table 4:** Comparison of cytokine/chemokine levels between human and PDX ascites using anti‐human or anti‐mouse antibodies

Cytokine/chemokine	Human PMP (human ab) ascites, median (range)^a^	PDX PMP (mouse ab) ascites, median (range)^b^	Significance
IP‐10	1610 (29.98–4883)	232 (28.17–1930)	ns
IL‐6	915 (28–9261)	142.5 (27.24–860)	0.0173
MCP‐1	838 (188–10,443)	204 (26.6–1494)	ns
IL‐8 (KC)	195 (107–938)	559.5 (121–1886)	ns
IL8 (MIP2)	195 (1.25–16.21)	62.74 (0.21–77.7)	0.0087
VEGF	103 (107–938)	66.28 (0.4–155)	ns
IL‐10	56.21 (1.66–337)	6.115 (1.21–75.14)	ns
MIP‐1*β*	18.63 (0.96–43.52)	45.53 (4.57–216)	ns
TNF‐*α*	9.09 (9.14–3792)	7.78 (21.7–6314)	ns
MIP‐1*α*	7.44 (2.03–30.92)	34.05 (10.9–119.3)	0.0173
IFN*γ*	3.02 (1.51–8.14)	0.95 (0.94–33.58)	ns

ns, not significant.

a Assays performed with anti‐human antibodies raised against listed antigens.

b Assays performed with anti‐mouse antibodies raised against listed antigens.

**Table 5:** Comparison of fold difference (FD) in median cytokine levels between human and PDX ascites using anti‐human (hab) and anti‐mouse antibodies (mab)

Cytokine/chemokine	FD hum ascites versus PDX ascites (hab)	FD hum ascites versus PDX ascites (mab)
IL‐6	897.06	6.42
IP‐10	86.93	6.94
IL‐8 (KC)	49.68	0.35
IL8 (MIP2)	49.68	0.35
IL‐10	33.86	9.19
MCP‐1	27.61	4.11
MIP‐1*β*	19.41	0.41
TNF‐*α*	7.27	1.17
MIP‐1*α*	3.67	0.22
IFN*γ*	0.54	3.18
VEGF	0.04	1.55

### Comparison of PMP human and PDX cytokine/chemokine profiles in sera

We next compared cytokine expression profiles of ascites and sera collected from PDX models in order to test if any of the 17 cytokines assayed show elevated expression in the mouse PDX sera. These studies did not reveal significant differences using either human or mouse antibodies (data not shown) suggesting that the cytokines that show increased expression in PDX ascites are restricted to the peritoneal tumor microenvironment. In addition, no significant elevation in the levels of the chemokines was seen in the sera of PDX models compared to ungrafted mice (data not shown) suggesting that none of the measured chemokines/cytokines could be used as a biomarker to track PMP tumor growth in these mouse models. C‐reactive protein (CRP), a biomarker commonly used in clinical practice to monitor disease progression, was also measured. As CRP is made by the liver, and is therefore, expected to be of murine origin in PDX mouse models, an anti‐mouse CRP antibody was used. CRP levels in PDX serum samples were not significantly different from endogenous CRP levels in mouse sera (data not shown). These results preclude the use of CRP as a biomarker to monitor PMP growth in PDX models.

## Discussion

Previous reports of generation of PDX mouse models of PMP described the close histopathological similarities between human PMP and PDX PMP 12, 13. These studies were the first reports of PDX mouse model generation for PMP and showed that human PMP tumors could be grown in a foreign environment such as the murine peritoneal cavity. Our studies extend these findings and show that PMP tumors in PDX models also show a similar chemokine/cytokine profile as their human counterparts. These results suggest that reciprocal interactions between tumor and tumor‐associated stromal components are also likely to be conserved in these PDX models and therefore, these models are good surrogates for modeling the PMP tumor microenvironment.

Mesothelial cells on the serosal surface of organs generate fluid that lubricates the movement of visceral organs against the peritoneal surface. This fluid is generally cleared through lymphatic drainage. In cancers that show a propensity to disseminate to the peritoneal cavity such as ovarian cancers and PMP, there is increased accumulation of peritoneal fluid (ascites) 24, 25, 26, 27. This often leads to bowel obstruction in advanced disease and, coupled with inflammation‐associated fibrosis, is the most frequent cause of mortality in PMP patients. Ascites accumulation is due to (1) secretions of tumor and associated stromal cells, (2) increased vascular permeability, and (3) decreased clearance due to tumor‐cell mediated lymphatic obstruction 24, 27. Therefore, the chemokine and cytokine secretions in the ascites provide a direct read‐out of the tumor microenvironment. However, sorting the relative contributions of tumor and stromal cells to chemokine/cytokine pools in ascites of human PMP can be complicated. The PMP PDX mouse models, in which the tumor cells are human‐derived and the stromal cells are mouse‐derived, provide a direct approach to the analysis of this problem.

In a recently published study, we reported the expression of a number of chemokines and cytokines in the ascites and sera from PMP patients. The resultant profile of the inflammatory peritoneal tumor microenvironment in PMP turned out to be distinct from that associated with pathogens (pathogen‐associated molecular pattern) or injury (danger‐associated molecular pattern) 15. The PMP mouse models that we have generated recapitulate not only the histopathology of the original PMP tumors (low and high grade) but also the chemokine/cytokine expression pattern of their human counterparts. Consistent elevations in IL6, IL8, IP10, MCP1, and MIP1*α* are seen in PDX mouse models as in human ascites. Our results suggest that these cytokines are not only produced by human‐derived PMP tumor cells, but also by mouse‐derived stromal cells.

Although many of the cytokines were elevated in ascites, none of them showed significant elevation in sera of PMP PDX mouse models. These results are consistent with what was seen in human PMP patients; all the cytokines/chemokines that were assessed showed significant enrichment in patient ascites but not in sera. These results, considered together, suggest that the peritoneal tumor microenvironment in PMP is partitioned from systemic circulation. The reasons for, or the nature of, this partition (physical or biochemical) is not clear. PMP tumors are derived from mucin‐producing goblet cells of the appendix and the mucin to cell ratio can be as high as 1000 to 1 1. Excessive extracellular mucin secretion could in part, restrict access or limit secretion of chemokines/cytokines to niches within the tumor microenvironment. It is tempting to speculate that the proximal utility of these chemokines and cytokines that show elevated expression in the ascites is to regulate tumor‐stromal communication and therefore, are bound by receptors on their respective cell surfaces and endocytosed soon after. This hypothesis remains to be tested.

A number of different stromal cell types have been identified and shown to alter tumor hallmark capabilities such as sustained proliferation, and initiation of angiogenesis, invasion and metastasis and evasion of (1) growth suppressors, (2) immune surveillance, (3) replicative senescence, and (4) cell death 28. These cell types include angiogenic vascular cells (endothelial cells and pericytes), infiltrating immune cells (CD4 and CD8 positive T cells, T regulatory cells, B cells, natural killer T (NK/T) cells, macrophages, inflammatory monocytes, neutrophils, mast cells, platelets), and cancer‐associated fibroblasts (mesenchymal stem cells, activated myofibroblasts, and adipocytes) 29. As noted before, athymic nude (Foxn1nu) mice were used as hosts for serial propagation of PMP tumors and generation of PDX models. Although these mice lack mature T cells, functional B cells, dendritic cells, macrophages, NK cells, and complement system are still present 30. Thus, these mouse models may not replicate the full gamut of immune and inflammatory responses seen in human PMP patients. In spite of this limitation, due to the close similarity in the pattern of chemokine/cytokine expression and histopathology, these PDX mice are strong preclinical models for PMP.

The human tumors that were implanted in the peritoneal cavities of mice are expected to contain tumor‐associated stromal components. Therefore, it is possible that some of these stromal cells of human origin may have contributed to the elevation of cytokines such as IL6, IL8 and GRO. This remains unlikely due to two reasons. First, tumor‐associated stromal cells are not expected to survive the serial passaging procedures that we have adopted as these cells are not transformed or immortalized as the tumor cells. Second, in the unlikely event of the survival of human stromal cells, the cytokines secreted by these cells would have cross‐reacted with human antibodies. If so, this would be at odds with the large difference in median values between PDX‐ and human PMP‐derived IL6, IP10, IL8, IL10, MCP1, MIP1*β*, TNF*α*, and MIP1*α* detected using antibodies raised against these human antigens. The fact that the fold differences in median levels of all these cytokines/chemokines were reduced when mouse antibodies were used support the notion that these cytokines were mostly produced by murine tumor‐associated stromal cells.

The cytokines that show elevated expression in human PMP and PDX ascites such as IL6, IP10, IL8, IL10, MCP‐1, and MIP‐1*β* have all been shown to perform autocrine and paracrine functions and regulate tumor–stromal cross talk. Colon cancer cells can stimulate macrophages to secrete IL6 which in turn, activates the IL6 receptor and STAT3 in tumor cells 31, 32. IP10, a critical regulator of interferon response, is secreted by leukocytes, activated neutrophils, monocytes, and fibroblasts and has been shown to regulate tumor growth and angiogenesis 33. IL‐8 has been implicated in regulation of metastasis, chemoresistance and angiogenesis 34. IL10, an anti‐inflammatory cytokine, secreted mostly by macrophages and T regulatory cells, has been shown to regulate T‐cell response and tumor immunity 35. MCP1, a monocyte chemoattractant, is important for recruitment of macrophages to the tumor microenvironment 36. As many of these cytokines and chemokines often perform antagonistic tumor‐promoting and tumor‐inhibiting functions, prediction of therapeutic benefits of inhibition of these proteins is not straight forward. The use of PDX mouse models to test the effectiveness of anti‐cytokine or anti‐chemokine antibodies (such as anti‐IL6 antibody) in reducing tumor growth is likely to become prevalent and our results suggest a note of caution in the design and interpretation of these studies. These studies will be not only confounded by seemingly contradictory roles of the target proteins in cancer development but also by the fact that some of these cytokines are produced by mouse‐derived stroma and therefore, the therapeutic agents would have to target mouse antigens.

Future studies will focus on defining the identities of stromal cell types in the peritoneal microenvironment and the relative contributions of these cell types to PMP pathobiology. Coculture studies of stromal components and PMP cell lines that we have generated in our laboratory (M. R. K., P. T., B. W. L., V. G., unpubl. results) will be useful in these investigations. In addition, the PMP PDX models will be excellent preclinical tools for testing the efficacy of anticancer agents and developing novel treatment interventions for PMP.

## Conflict of Interest

None declared.
